# Development of Graphene Oxide-Based Nonedible Cottonseed Nanofluids for Power Transformers

**DOI:** 10.3390/ma13112569

**Published:** 2020-06-04

**Authors:** Rizwan A. Farade, Noor Izzri Abdul Wahab, Diaa-Eldin A. Mansour, Norhafiz B. Azis, Jasronita bt. Jasni, Manzoore Elahi M. Soudagar, Vasudevamurthy Siddappa

**Affiliations:** 1 Department of Electrical and Electronics Engineering, Faculty of Engineering, University Putra Malaysia, Serdang 43400, Malaysia; norhafiz@upm.edu.my (N.B.A.); jas@upm.edu.my (J.b.J.); 2 Department of Electrical Power and Machines Engineering, Faculty of Engineering, Tanta University, 31512 Tanta, Egypt; mansour@f-eng.tanta.edu.eg; 3 Department of Mechanical Engineering, Faculty of Engineering, University of Malaya, Kuala Lumpur 50603, Malaysia; me.soudagar@gmail.com; 4 Department of Electrical and Electronics Engineering, Dr. Ambedkar Institute of Technology, Bengaluru 560056, India; vasudevamurthy.s@dr-ait.org

**Keywords:** transformer oil, nanofluid, graphene oxide, dielectric properties, thermal properties

## Abstract

Sustainable materials, such as vegetable oils, have become an effective alternative for liquid dielectrics in power transformers. However, currently available vegetable oils for transformer application are extracted from edible products with a negative impact on food supply. So, it is proposed in this study to develop cottonseed oil (CSO) as an electrical insulating material and cooling medium in transformers. This development is performed in two stages. The first stage is to treat CSO with tertiary butylhydroquinone (TBHQ) antioxidants in order to enhance its oxidation stability. The second and most important stage is to use the promising graphene oxide (GO) nanosheets to enhance the dielectric and thermal properties of such oil through synthesizing GO-based CSO nanofluids. Sodium dodecyl sulfate (SDS) surfactant was used as surfactant for GO nanosheets. The nanofluid synthesis process followed the two-step method. Proper characterization of GO nanosheets and prepared nanofluids was performed using various techniques to validate the structure of GO nanosheets and their stability into the prepared nanofluids. The considered weight percentages of GO nanosheets into CSO are 0.01, 0.02, 0.03 and 0.05. Dielectric and thermal properties were comprehensively evaluated. Through these evaluations, the proper weight percentage of GO nanosheets was adopted and the corresponding physical mechanisms were discussed.

## 1. Introduction

Insulating fluids based on mineral oil are used in oil-immersed power transformers. Mineral oil is a low-cost fluid that mostly accomplishes dual purposes, as a successful dielectric material and as a cooling medium. However, the sustainable development of transformer oil is heatedly under debate in the world, because of the limitation of the resources of mineral oil, which mainly are based on petroleum products. In addition, mineral oil has serious deficiencies such as, low biodegradability (<30%), low flash and fire points, low moisture saturation limit, and noncompliance with health or environmental laws [1].

Such challenges have intensified the interest for alternative solutions that can be manufactured from resources readily available and that could provide the greatest and potential long-term prospects. One of the most promising solutions is to use vegetable oils in the form of natural esters. Many researchers and industries considered natural esters as insulating oils in power transformers [2,3]. 

Currently, there are many transformer grade commercial esters. These include natural esters like BIOTEMP^®^, Envirotemp^®^FR3^TM^, MIDEL 1204, and MIDEL 1215, or synthetic esters like MIDEL 7131. The differences between these types are mainly attributed to the source from which they have been extracted if they are natural esters or attributed to the constituents if they are synthetic esters. For mentioned natural esters, they have been extracted from sunflower, soya, rapeseed, and so on. These differences between various types are reflected on various properties, such as density, viscosity, thermal conductivity, and breakdown voltage (BDV). Most currently existing natural esters are formulated and typically contain a minimum 95% of base vegetable oil with the remaining percentage as additives. These additives are generally applied for improved properties, according to IEEE Standard C57.147-2018 [4], which lists the acceptable values for various properties of natural ester insulating fluids. In addition to these properties, these esters proved beneficial performance considering some practical issues. For example, the dielectric strength Envirotemp^®^FR3^TM^ and MIDEL 7131 was less affected by the presence of particles, either cellulose particles or copper particles [5]. Also, papers impregnated in Envirotemp^®^FR3^TM^ and MIDEL 7131 had higher tensile strengths than that impregnated in mineral oil under various aging conditions [6,7]. On the other hand, MIDEL 1204 and MIDEL 1215 could prove lower annual percent loss of life compared to mineral oil, while MIDEL 7131 could prove the lowest annual percent loss of life among all [3].

In spite of vegetable oils could provide better properties as a transformer insulating oil; their commercial application faces some challenges. These challenges include the existence of unsaturated fatty acids and the extraction from edible products. The existence of unsaturated fatty acids makes vegetable oils unstable and vulnerable for oxidation with a negative impact on various oil properties. To overcome this challenge, some additives were used with vegetable oils as antioxidants to enhance the critical properties of oil, especially under thermal aging conditions [8,9]. On the other hand, the extraction from edible products will consume these products for insulating fluid applications with a negative impact on food supply.

In India, it is projected to be the fastest-growing markets in the energy sector, in the future including the development of transmission and distribution (T&D) infrastructure. This development is reflected on the growing demand for power transformers, with a driving market for transformer oil. On the other hand, India is the world’s second largest cotton producer [10], making cottonseed oil (CSO) readily available as nonedible oil. So, there is an excellent opportunity in India to convert this nonedible CSO feedstock into commercial grade insulating fluid for applications in transformers. 

CSO is biodegradable and nontoxic, making it environmentally friendly [11]. In addition, it has high flash and fire points, making it less flammable than mineral oil. However, like other natural esters, it has less saturated fatty acids compared to unsaturated ones, resulting in greater tendency towards oxidation. This oxidation tendency can be overcome by upgrading to a higher oleic content [12] or by adding antioxidants. Very few researchers discussed the use of CSO in transformers as an alternative insulating fluid and demonstrated its compatibility with transformer parts. In addition, the acid and copper strip corrosion values for CSO were found to be within the standard specifications [13]. However, CSO demonstrated lower BDV than other natural esters [14].

Recently, the introduction of nanoparticles was proposed to modify insulating fluids and prepare what is called nanofluids in order to improve thermal and dielectric properties [15,16,17,18]. The performance of nanofluids depends primarily on the filling percentage, particle type, size, shape and the agglomeration rate [19,20,21,22,23]. The nanofillers are generally classified into metals, metal oxides and carbon-based substances. Using metal oxides as nanofillers could improve the dielectric properties of the base fluids [24,25]. With respect to carbon-based nanofillers, they could improve thermal properties of the base fluids. Carbon nanotubes (CNTs) are coming at the forefront of these nanofillers, even at low filler percentage [26]. These nanofillers have single-dimensional morphology with Van der Waals π–π stacking interfaces, accelerating agglomeration between CNT fibrils. To avoid the aggregation of CNTs, they must be functionalized with strong acids, which induces accidental defects in uninterrupted sp2-bonded structures of carbon. Such defects increase the dispersion of the phonon, which greatly quenches the thermal conductivity [27,28].

In order to overcome such gridlocks, the researchers have been motivated to develop two-dimensional (2D) structures like graphene [29,30] and grapheme oxide (GO): a 2D hexagonal carbon lattice. The integrated sp2 covalent bonding of graphene carbon atoms creates an extraordinarily high thermal conductivity [31]. In addition, the 2D carbon material nature contributes to elevated electrical insulation [32], making them an attractive nanofillers for improving the transformer oil overall properties.

Based on the challenges in the currently available natural esters as edible sources, this study aims to develop CSO for the application in power transformers. In addition, it is proposed to enhance the dielectric and thermal properties of such oil using the promising 2D GO nanofillers. First, CSO was treated with tertiary butylhydroquinone (TBHQ), and GO was modified with sodium dodecyl sulfate (SDS) surfactant. Then, GO-based cottonseed nanofluids were prepared. A detailed description of synthesis and characterization of GO and nanofluids was presented. Different weight percentages (wt%) of GO nanofillers were introduced into CSO, 0.01, 0.02, 0.03 and 0.05. Comprehensive dispersion stability analyses of CSNFs were performed. The dielectric and thermal properties of CSNFs were evaluated in comparison to base CSO. Finally, the governing mechanisms behind the obtained results were discussed.

## 2. Experimental Process

### 2.1. Materials and Characterization Techniques

GO was obtained in a powder form from Sigma Aldrich (Bangaluru, India). It has the following properties; molecular weight 4239.48 g/mol, 4–10% edge-oxidized, and 15–20 sheets. 

For the scale and morphological study of GO nanoparticles, Transmission Electron Microscope (TEM, JEM-2100-PLUS, JOEL, Tokyo, Japan) was used and operated with a 200 kV accelerating voltage. Phase purity identification of nanoparticles was authenticated by powder X-ray diffraction (XRD, Philips-X’pert-MPD, PANalytical, Almelo, Netherlands) with radiation Cu Kß in range (2Ɵ) 5–100°. Determination of the abundance of specific elements in GO nanoparticles is detected by Energy-Dispersive X-Ray Spectroscopy (EDX, JSM-IT 500, JOEL, Tokyo, Japan).

### 2.2. Preparation of Nanofluids

**Figure 1:**
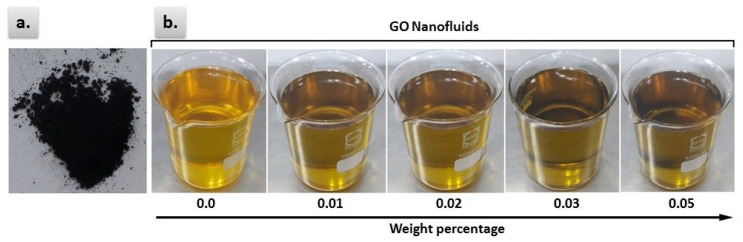
Visual images of the graphene oxide (GO) powder (**a**) and the prepared nanofluids (**b**).

The two-step method is used for preparing nanofluids, taking into account its economical and productive features over the one-step method [33]. The CSO was delivered by the local Indian vendor (M/s S.S. Odunavar Industries, Laxmeshwar, India) and TBHQ antioxidant was purchased from Sigma Aldrich (Bangaluru, India). Initially calculated amount of TBHQ antioxidant is added to CSO. The solution was magnetically stirred for 1 h at 80 °C. The considered weight percentage of GO and the corresponding amount of surfactant SDS were added to this solution and the mixture was stirred again for 30 min at the same temperature. Finally, GO nanoparticles were dispersed by a Probe Sonicator (Samarth Electronics, Ambarnath (E), India) for a period of 1 h [34]. The considered weight percentages of GO nanoparticles are 0.01 wt%, 0.02 wt%, 0.03 wt% and 0.05 wt%. Figure 1 includes the visual images of the GO powder and the prepared nanofluids at different weight percentages.

### 2.3. Measurement Techniques

The measured dielectric properties of nanofluids are BDV, dielectric constant, loss tangent, resistivity and capacitance. For BDV, it was measured at room temperature in accordance with the standard IEC 60156. 10 breakdowns were performed for each sample in order to obtain reliable results. Dielectric properties were measured by ELTEL (ADTR-2K PLUS, ELTEL Industries, Bangaluru, India) instrument (500 V AC and 50 Hz for dielectric constant, loss tangent and capacitance testing and 500 V DC for resistivity testing) at four distinct temperatures (45, 60, 75 and 90 °C). For dielectric properties, the measurements were repeated only 3 times for each sample, since there is no significant deviation between measurements obtained for the same sample.

**Figure 2:**
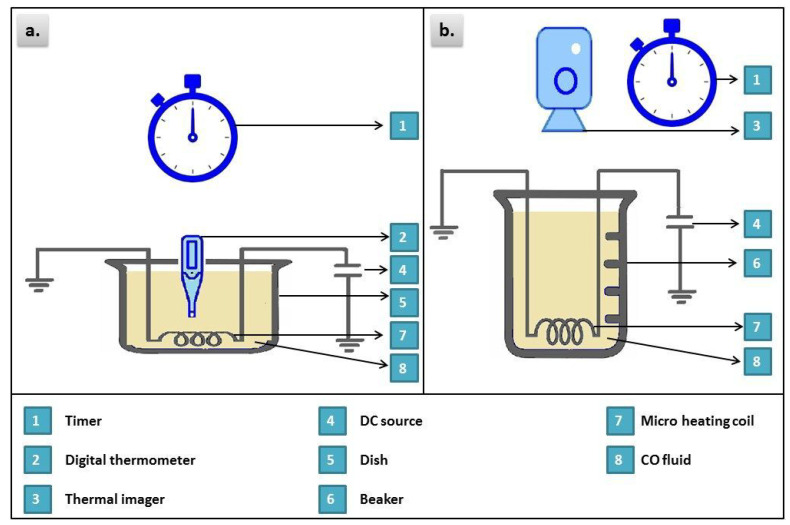
Experimental setup for thermal response (**a**) and surface temperature thermogram (**b**).

The measured thermal properties of nanofluids are thermal conductivity, thermal response, and thermogram. For the thermal conductivity, it has been tested using a Decagon instrument (KD2-PRO, Decagon Devices Inc., Pullman WA, USA) by a transient hot-wire technique. Measurements were performed at four different temperatures (35, 45, 55 and 65 °C). For thermal response test, 100 mL of the specimen of the considered sample was placed in a container as shown in Figure 2a. The specimen was heated through an electrical heating coil for 26 min, and then it was allowed to cool down naturally for a further 26 min. The heating and cooling rates were recorded. For thermogram test, the considered sample was heated in a beaker for 45 min and the surface temperature images were recorded as shown in Figure 2b for different time intervals using Fluke Ti125 thermal imager.

## 3. Structural Characterization and Stability Analysis

### 3.1. Structural Characterization

**Figure 3:**
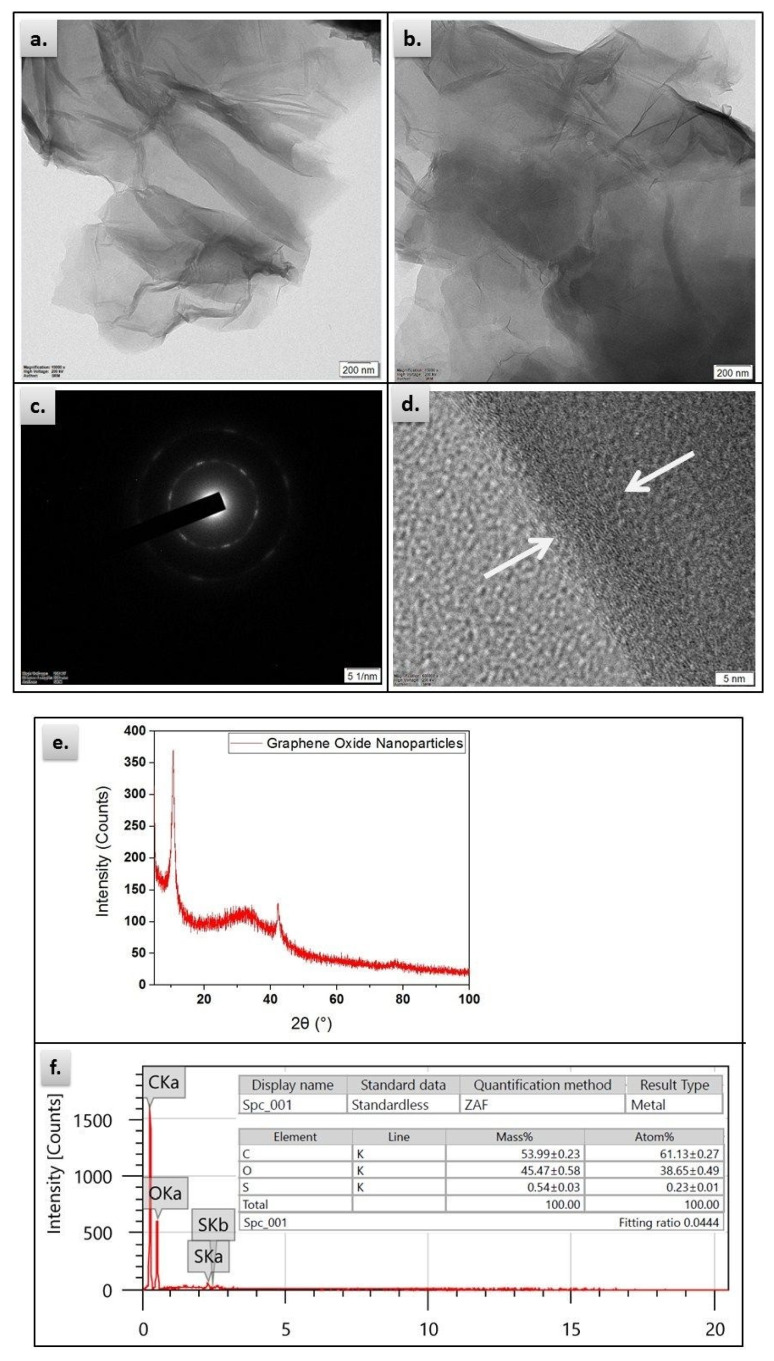
Characterization of GO powder: Transmission Electron Microscope (TEM) analysis at magnifications: (**a**) 1000× (**b**) 1500×; (**c**) selected area diffraction (SAED) pattern; (**d**) the edge area of the GO, indicating sheets consists of a few stacked atomic layers; (**e**) powder X-ray Diffraction (XRD); and (**f**) energy dispersive X-ray (EDX) spectroscopy.

TEM imaging was obtained by dispersing GO powder in an ethanol, dropping on a copper grid, and drying under vacuum for 24 h. The obtained TEM images are illustrated in Figure 3a,b at two separate magnification levels, having a smooth and wrinkled surface. The images had different transparencies along the whole area indicating a sheet like morphology similar to that observed in [35]. The higher the transparency, the fewer number of stacked GO layers. From the selected area diffraction image for GO in Figure 3c, one array of hexagonal diffraction pattern was observed as a characteristic feature for GO. Also, Figure 3d shows the edge area of the GO, in which each sheet consists of stacked atomic layers, where each fringe depicts one atomic layer. 

XRD pattern of GO is illustrated in Figure 3e. The patterns are realized in the 2-theta (2θ) range from 0 to 100°. The XRD pattern was recorded at speed 50° per minute at step width 0.01°. The crystalline structure is evident from the pattern and a signature peak around 10.5° is indicated that depends upon the amount of oxygen in GO [36,37]. GO EDX is shown in Figure 3f. It shows the chemical elements in the sample. The amount of carbon that was found in this test is relatively large (Mass% 53.99, Atom% 61.13) followed by oxygen (Mass% 45.47, Atom% 38.65), while the impurities are fractional (Mass% 0.54, Atom% 0.23).

### 3.2. Stability Analysis of GO-Based CSO Nanofluids

Stability analysis of GO-based CSO nanofluids are performed using UV–Vis optical spectroscopy over a wavelength ranging between 200 nm and 800 nm. Since the SDS surfactant plays the critical role in stabilization of GO nanoparticles, five different GO:SDS ratios are considered (1GO:1SDS, 1GO:2SDS, 1GO:3SDS, 1GO:4SDS, and 1GO:5SDS).

**Figure 4:**
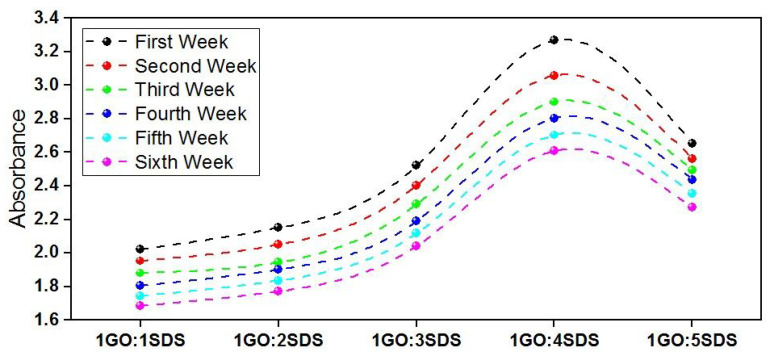
Absorbance of CO-based various ratios of GO:SDS samples over a period of six weeks.

Stability analysis was performed over a period of six weeks with recording the absorbance every week. For all samples, the UV-Vis spectra peaked at a wavelength of 250 nm, which was closer to that obtained with other GO nanofluids [38]. Figure 4 depicts the obtained results. For each GO:SDS ratio, a slight reduction in the absorbance occurred indicating that few GO nanoparticles were settling down. After the first week period, maximum absorbance was recorded for 1GO:4SDS ratio and minimum absorbance was recorded for 1GO:1SDS. But, after the sixth week, the absorbance drop of 1GO:4SDS ratio was about 17%, which is higher than that of other ratios. This can be attributed to the formation of double chain with the excess ratio of surfactant [39]. The lowest absorbance drops were observed in 1GO:1SDS and 1GO:5SDS. To keep lower acid content into the oil, 1GO:1SDS ratio was adopted for the current implementation.

## 4. Dielectric Properties of GO-Based CSO Nanofluids 

### 4.1. Breakdown Voltage

**Figure 5:**
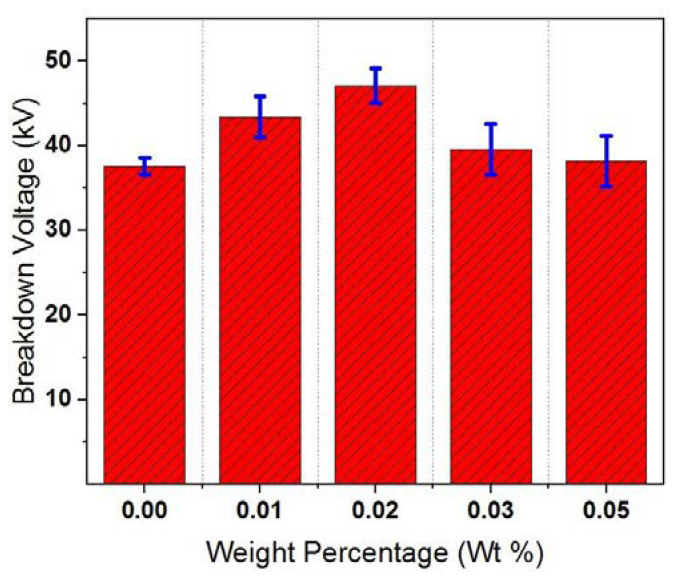
AC Breakdown voltage of GO-based cottonseed oil (CSO) nanofluids at different weight percentages.

The electrical breakdown property of insulating fluids indicates its capability to withstand high voltages. Consequently, higher breakdown voltages are needed. The mean AC BDV of TBHQ CSO was measured as 37.6 kV after degassing. After adding GO nanoparticles, AC BDV was enhanced as shown in Figure 5. At 0.02 wt% of GO loading percentage, the enhancement attainted its maximum value of about 25.3% in comparison to bare TBHQ CSO. Further increase of GO loading percentage to 0.03 wt% and 0.05 wt% caused declining results. The mechanisms behind these results can be explained as follows.

**Figure 6:**
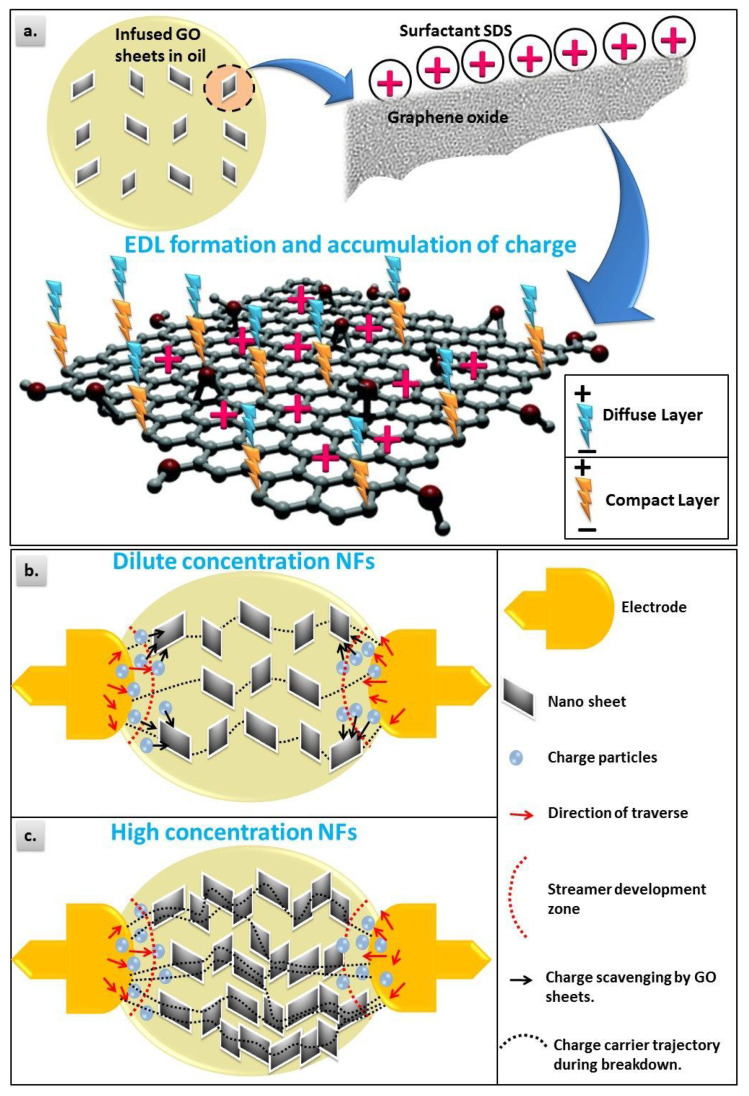
Physical mechanisms behind breakdown strength of nanofluids. (**a**) Electrical double layer (EDL) formation; (**b**) capturing and trapping electrons at low GO weight percentages; (**c**) overlap between adjacent EDLs at high GO weight percentages.

According to the theory of electrical double layer (EDL) [40], the surface of nanoparticles in contact with transformer oil will accumulate free static charges. These static charges attract the counter-ions in the transformer oil and repel co-ions. Near the surface of the charged nanoparticles, there is a counter-ion film that is strongly bound to the surface of the nanoparticles. This layer is referred to as the compact layer of EDL as shown in Figure 6a. The net charge density decreases progressively to zero while moving towards the oil volume. In this region, ions are less strongly bounded and can move under electrostatic forces. This area is regarded as the diffuse layer of EDL. Accordingly, EDL acts as traps that capture the brisk electrons produced under high electric field as illustrated in Figure 6b. Thus, EDL plays the significant role in enhancing the breakdown characteristics of nanofluids [41]. 

Following capturing of electrons, they are stored on the GO nanosheets [42]. The charge saturation on nanoparticles depends on the surface area [43], which is significantly large in GO nanosheets due to 2D structure. Due to the homogeneous distribution of NPs in the oil, the EDL volume predominates and plenty of traps will appear in the system. As a result, the BDV of oil increases with growing filler concentration.

Conversely, higher concentration of GO nanosheets above the critical concentration (0.02 wt% in the present study) contributes to an overlap between adjacent EDLs causing a reduction of the overall EDL volume as shown in Figure 6c. This decreases the trap sites with a subsequent decrease in electron capturing. Therefore, the BDV reduces after this critical concentration. 

### 4.2. Dielectric Constant, Loss Tangent and Resistivity

As an electrical insulating oil, there are three important dielectric properties that need to be investigated to monitor the oil quality. These properties are dielectric constant, loss tangent and resistivity. The dielectric constant corresponds to the lack of ability of molecules in the dielectric to reorient with an alternating electric field gradient. The loss tangent (tan δ) indicates the loss of energy into the dielectric. Whereas resistivity is an intrinsic material feature that indicates how strongly a certain material resists the flow of electric current.

**Figure 7:**
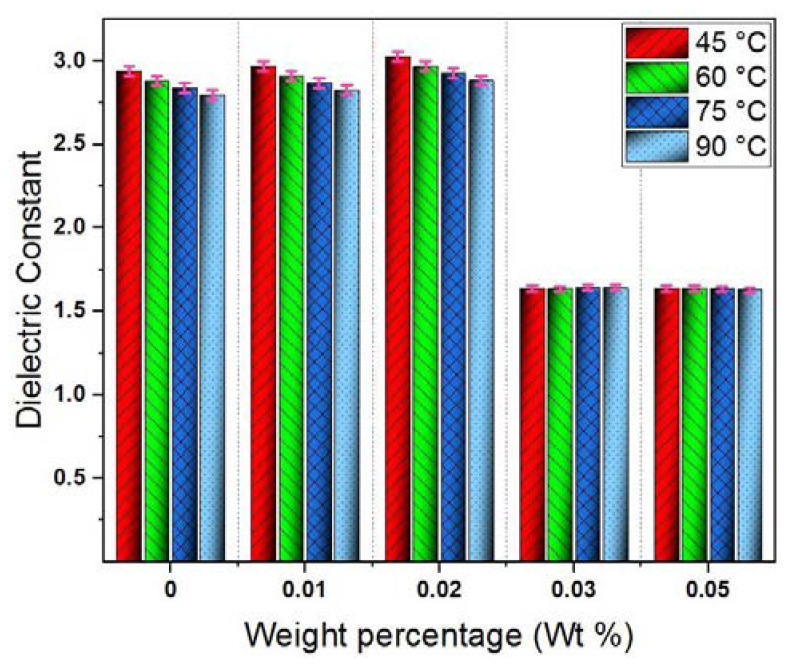
Dielectric constant of GO-based CSO nanofluids against weight percentage at various temperatures.

The permittivity of dielectric material under alternating electrical field is a complex parameter and is denoted as follows:

(1) $$\varepsilon^{*}=\varepsilon^{\prime }-\;i\varepsilon^{\prime \prime }$$

where the real part ε′ is the dielectric constant and the imaginary part $\varepsilon^{\prime \prime }$ is called the loss factor. The dielectric constant of nanofluids is presented in Figure 7. Regarding its dependence on GO weight percentages, it indicates a slight increase against weight percentage up to 0.02 wt%, followed by a remarkable decrease at 0.03 wt% and 0.05 wt%. This trend can be explained considering the polarizability of liquid/solid mixtures. 

According to classical theories, this polarizability is composed of two terms, polarizability of liquid molecules and inner polarizability of solid particles. This makes the composite relative permittivity (ε) to be given as follows [44]:

(2) $$\frac{\varepsilon -1}{\varepsilon +2}=\frac{1}{{3\varepsilon }_{0}}\left[N_{1}\alpha_{1}{+N}_{2}\alpha_{2}\right]$$

where N_1_ and N_2_ are the number of liquid and solid molecules per unit volume, respectively, while α_1_ and α_2_ are their corresponding polarizabilities. But, at the nanoscale, the situation becomes different, where the EDL at nanoparticle/oil interface will have different polarizability (α_3_) rather than the above two terms. This polarizability is limited due to rigid structure created at the compact layer [41,45]. The number of liquid molecules at EDL are then deducted from the total number of liquid molecules (N_1_) and a modified equation for the composite relative permittivity is proposed as follows:

(3) $$\frac{\varepsilon -1}{\varepsilon +2}=\frac{1}{{3\varepsilon }_{0}}\left[\left(N_{1}{-N}_{3}\right)\alpha_{1}{+N}_{2}\alpha_{2}{+N}_{3}\alpha_{3}\right]$$

where N_3_ is the number of liquid molecules at EDL per unit volume, and thus, (N_1_ − N_3_) represents the remaining number of liquid molecules (oil molecules other than nanoparticle/oil interface) in the free liquid space. 

At low GO weight percentage (0.01 wt% and 0.02 wt%), nanofluids had a slightly higher relative permittivity than that of pure CSO as shown in Figure 7. At this low weight percentage, the number of liquid molecules at EDL is small and has a negligible effect. Thus, the total polarization increases due to the increase in the number of GO nanosheets that have higher polarizability than oil molecules. 

At higher GO weight percentage (0.03 wt% and 0.05 wt%), the number of liquid molecules at EDL increased significantly, resulting in a decrease in the number of remaining liquid molecules in the free liquid space. Considering the limited polarizability at EDL, the increase in the third term of Equation (3) due to increase in N_3_ will be small. But, the first term decreases significantly due to increase in N_3_. Therefore, the overall effect will be a decrease in the dielectric constant as obtained in Figure 7. 

**Figure 8:**
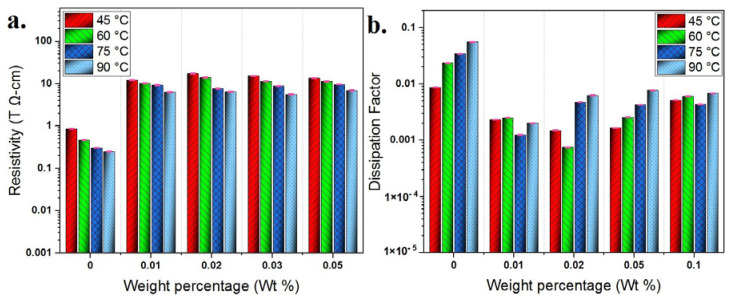
Variation of (**a**) resistivity and (**b**) dissipation factor against weight percentage at various temperatures.

For the resistivity and loss tangent, they are depicted in Figure 8 at various GO weight percentages and various temperatures. The resistivity in Figure 8a increases against weight percentage up to 0.02 wt%. At 0.02 wt% and 45 °C, the resistivity attained 17.34 TΩ-cm compared to 0.8613 TΩ-cm for the base CSO at the same temperature. Above this weight percentage, either negligible increase or decrease in the resistivity is observed depending on the temperature. This resistivity dependence on weight percentage aligns with that obtained with BDV. 

For the loss tangent, it is also referred to as the dissipation factor and is defined in terms of the imaginary and real parts of permittivity as follows:

(4) $$tan\;\delta =\frac{\varepsilon^{\prime \prime }}{\varepsilon^{\prime }}=\frac{I_{l}}{I_{c}}$$

where δ is the loss angle between the effective current *I_l_* and the charging current *I_c_*. Figure 8b indicates that the dissipation factors at 45 °C and 60 °C decreased against the weight percentage up to 0.02 wt%, before increasing again above this weight percentage. Similar trends were obtained at 75 °C and 90 °C with shifting the lowest point to be at 0.01 wt%.

The increase in resistivity and the decrease in dissipation factor is attributed to the role of EDL in capturing and trapping charge carriers and storing them on the surface of GO nanosheets. For lower temperatures (45 °C), resistivity enhancements peak at 0.02 wt%, where above this weight percentage overlaps between EDLs enabling few transitions of charge carriers between adjacent EDLs. At higher temperatures (90 °C), the peak enhancements shifted slightly to be at 0.01 wt%. This is attributed to the Brownian motion of GO nanosheets, which increases with the temperature rise [46], making it is possible for EDLs to overlap at lower weight percentage. But generally, the difference between 0.01 wt% and 0.02 wt% is small and both weight percentages exhibited significant enhancements. So, by considering these properties together with BDV, 0.02 wt% is preferred.

## 5. Thermal Properties of GO-Based CSO Nanofluids

### 5.1. Thermal Conductivity and Thermal Response

**Figure 9:**
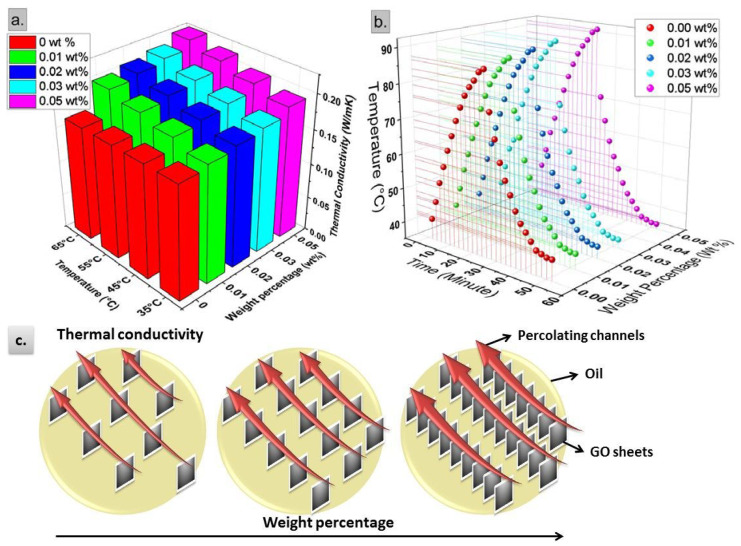
Thermal conductivity enhancements in GO-based CSO nanofluids. (**a**) Thermal conductivity; (**b**) thermal response; (**c**) proposed heat transport mechanism.

For cooling purposes of insulating fluids, it is important to enhance their thermal conductivity. In the present study, GO nanosheets could effectively fulfill this aim as shown in Figure 9. 

Figure 9a displays the thermal conductivity of GO-based CSO nanofluids against both weight percentage and temperature. In contradictory to dielectric properties, the thermal conductivity continuously increases with increasing the GO weight percentage without any decrement up to the maximum considered weight percentage of 0.05 wt%. The highest enhancement at 35 °C was 15.8% compared to the base CSO at the same temperature. From Figure 9a, it is also clear that the enhancement in thermal conductivity for nanofluids is temperature-dependent, in contradictory to the base CSO, which exhibited approximately flat characteristics against temperature. The highest enhancement at 65 °C was 36.4% compared to the base CSO. So, nanofluids behave as smart fluids capable to dissipate more heat at higher temperatures. 

The enhancement in thermal performance of nanofluids was validated through the thermal response results depicted in Figure 9b. Nanofluids had faster rate of heat transport either during heating up or during cooling down. This effectiveness was boosted with increasing the weight percentage of GO nanosheets.

Based on the obtained enhancements in thermal performance of GO-based CSO nanofluids, the following mechanisms are proposed as the governing ones: (1) phonon transport through GO nanosheets, (2) Brownian motion of GO nanosheets, and (3) interparticle interactions due to EDL. 

Regarding phonon transport, it is represented by a quanta of lattice vibrations. In graphene, there are plenty of phonon group due to the strong covalent sp^2^ bonding [47]. The behaviour of phonons depends on their mean free path (MFP) in comparison to the nanosheet thickness of graphene. To get enhanced heat transport, the phonon MFP should be larger than the nanosheet thickness to enable heat transport based on ballistic phonons [48]. For graphene, the phonon mean free path is in the order of 800 nm [49], which is larger than the nanosheet thickness. So, the ballistic phonons contribute to the heat transport process. Increasing the temperature leads to faster motion and vibration of the atoms within the nanosheets, thereby increasing the number of phonons. The thermal conductivity term according to phonon transport (*K_p_*) can be expressed as [47]:

(5) $$K_{p}=\overset{n}{\underset{i=1}{\sum }}C_{i}\left(\omega \right)v_{i}^{2}\left(\omega \right)\tau_{i}\left(\omega \right)d\omega$$

Where, *i* is the branch index, *n* is the number of phonon polarization branches, *ω* is the phonon frequency, *C_i_* is the mode specific heat, *v_i_* is the phonon group velocity, and *τ_i_* is the relaxation time. 

The second mechanism behind enhancements in thermal performance of GO-based CSO nanofluids is Brownian motion [46]. Brownian motion is caused by micro convection within the fluid. It was confirmed that Brownian motion is responsible for the abnormal increase in thermal conductivity of nanofluids [50,51,52]. The thermal conductivity term according to Brownian motion (*K_Br_*) can be expressed as [53]:

(6) $$K_{Br}=\frac{NL_{np}C_{V}}{3}\frac{K_{B}T}{3\pi \mu D_{np}L_{oil}}$$

Where, *N* is the concentration of nanoparticles, *L_np_* is the MFP of nanoparticles, *C_V_* is the specific heat, *K_B_* is Boltzmann constant, *T* is the temperature, *μ* is the viscosity, *D_np_* is the nanoparticle diameter, and *L_oil_* is the MFP of oil molecules. It is clear that temperature rise effectively increases Brownian motion-based thermal conductivity as a result of the increase in Brownian movement of nanoparticles.

The last contributed mechanism toward enhanced thermal performance of GO-based CSO nanofluids is the inter-particle interactions due to EDL. The repulsion Coulomb force caused by EDL adds further movement to nanoparticles with a subsequent enhancement in thermal conductivity. The thermal conductivity term according to EDL (*K_EDL_*) can be expressed as [53]:

(7) $$K_{EDL}=\frac{NL_{np}C_{V}}{3}\sqrt{\frac{Aq^{2}L_{np}}{\varepsilon_{oil}M_{np}{\left(r\varphi^{-1/3}\right)}^{2}}}$$

where *A* is the Coulomb constant, *q* is the surface charge of nanoparticles, *ε_oil_* is the dielectric constant of oil, *M_np_* is the nanoparticle mass, *r* is the nanoparticle radius, and *φ* is the nanoparticle volume fraction. In contradiction to dielectric properties, EDL overlapping at high GO weight percentages additionally enhances thermal conductivity due to formation of percolating channels for heat transport [54].

All above-mentioned mechanisms with conventional Maxwell thermal conductivity contribute to the overall thermal conductivity of GO-based CSO nanofluids. As a result, the overall effect is continuous increase in the thermal conductivity against GO weight percentage as shown in Figure 9c, even at high weight percentages (0.03 wt% and 0.05 wt%). Here, the temperature rise causes an increase in the percentage enhancement of thermal conductivity due to the temperature impact on phonon transport and Brownian motion as discussed above.

### 5.2. Thermogram Analysis 

**Figure 10:**
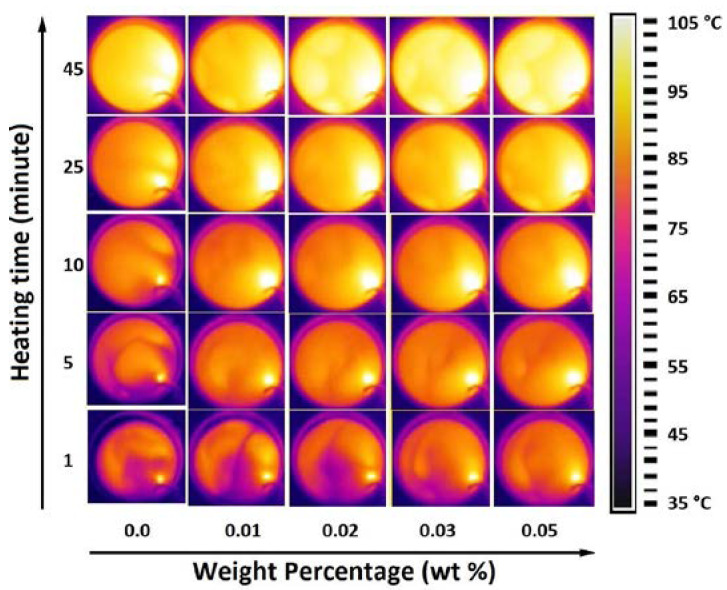
Thermogram analysis of GO-based CSO nanofluids with heating time against weight percentage.

Figure 10 displays the infrared images captured for GO-based CSO nanofluids in comparison to the base CSO. After heating for 45 min, it is clear that the surface temperatures of nanofluids are higher than that of the base fluid. The mean surface temperature for nanofluids was 102 °C at 0.05 wt%, while this temperature for the base oil was 95 °C. This validates the enhanced thermal conduction of nanofluids.

## 6. Conclusions

In this work, cottonseed nanofluids were developed as nonedible oil for insulating and cooling purposes in power transformers. The 2D GO nanosheets were used as the nanofillers and SDS was used as surfactant. Different weight percentages of GO nanosheets were used. For BDV, 10 breakdowns were performed for each sample, while for other dielectric properties, 3 measurements were taken for each sample. The main outcomes from this work can be summarized as follows:

1. Stability analysis of GO-based CSO nanofluids were performed using UV–Vis optical spectroscopy over a wavelength ranging between 200 nm and 800 nm. For all samples, the UV-Vis spectra peaked at a wavelength of 250 nm. The 1GO:1SDS ratio was adopted, where it exhibited the lowest absorbance drop.
2. The AC BDV has been enhanced by about 25% at GO weight percentage of 0.02 wt%. This enhancement was explained in terms of the role of EDL acts in traps and capturing the brisk electrons produced under high electric field.
3. The dielectric constant indicated a slight increase against weight percentage of GO nanosheets up to 0.02 wt%, followed by a remarkable decrease at 0.03 wt% and 0.05 wt%. This was explained considering polarizability of liquid molecules and inner polarizability of solid particles in addition to polarizability at EDL.
4. The resistivity has been increased and the dissipation factor has been decreased against weight percentage of GO nanosheets up to 0.02 wt%. This was attributed to the role of EDL in capturing and trapping charge carriers and storing them on the surface of GO nanosheets.
5. Above weight percentage 0.02 wt%, a decrement has been occurred in BDV and dielectric properties due to possible overlapping between adjacent EDLs, thereby reducing their effectiveness.
6. The thermal properties, investigated by thermal conductivity, thermal response and thermogram proved continuous enhancement in thermal performance against weight percentage up to the maximum considered weight percentage of 0.05 wt%.
7. Nanofluids behaved as smart fluids capable of dissipating more heat at higher temperatures, where the highest enhancement at 65 °C was 36.4% compared to 15.8% at 35 °C.
8. Phonon transport through GO nanosheets, their Brownian motion, and their interparticle interactions due to EDL were proposed as the governing mechanism behind thermal performance enhancement.
